# National Outbreak of *Salmonella* Serotype Saintpaul Infections: Importance of Texas Restaurant Investigations in Implicating Jalapeño Peppers

**DOI:** 10.1371/journal.pone.0016579

**Published:** 2011-02-23

**Authors:** Rajal K. Mody, Sharon A. Greene, Linda Gaul, Adrianne Sever, Sarah Pichette, Ingrid Zambrana, Thi Dang, Angie Gass, René Wood, Karen Herman, Laura B. Cantwell, Gerhard Falkenhorst, Kathleen Wannemuehler, Robert M. Hoekstra, Isaac McCullum, Amy Cone, Lou Franklin, Jana Austin, Kristin Delea, Casey Barton Behravesh, Samir V. Sodha, J. Christopher Yee, Brian Emanuel, Sufian F. Al-Khaldi, Val Jefferson, Ian T. Williams, Patricia M. Griffin, David L. Swerdlow

**Affiliations:** 1 Scientific Education and Professional Development Program Office, Epidemic Intelligence Service, Centers for Disease Control and Prevention, Atlanta, Georgia, United States of America; 2 Division of Foodborne, Waterborne, and Environmental Diseases, National Center for Emerging and Zoonotic Infectious Diseases, Centers for Disease Control and Prevention, Atlanta, Georgia, United States of America; 3 Texas Department of State Health Services, Austin, Texas, United States of America; 4 United States Food and Drug Administration, Silver Spring, Maryland, United States of America; 5 Wichita Falls-Wichita County Public Health District, Wichita Falls, Texas, United States of America; 6 National Center for Environmental Health, Centers for Disease Control and Prevention, Atlanta, Georgia, United States of America; Aga Khan University, Pakistan

## Abstract

**Background:**

In May 2008, PulseNet detected a multistate outbreak of *Salmonella enterica* serotype Saintpaul infections. Initial investigations identified an epidemiologic association between illness and consumption of raw tomatoes, yet cases continued. In mid-June, we investigated two clusters of outbreak strain infections in Texas among patrons of Restaurant A and two establishments of Restaurant Chain B to determine the outbreak's source.

**Methodology/Principal Findings:**

We conducted independent case-control studies of Restaurant A and B patrons. Patients were matched to well controls by meal date. We conducted restaurant environmental investigations and traced the origin of implicated products. Forty-seven case-patients and 40 controls were enrolled in the Restaurant A study. Thirty case-patients and 31 controls were enrolled in the Restaurant Chain B study. In both studies, illness was independently associated with only one menu item, fresh salsa (Restaurant A: matched odds ratio [mOR], 37; 95% confidence interval [CI], 7.2–386; Restaurant B: mOR, 13; 95% CI 1.3–infinity). The only ingredient in common between the two salsas was raw jalapeño peppers. Cultures of jalapeño peppers collected from an importer that supplied Restaurant Chain B and serrano peppers and irrigation water from a Mexican farm that supplied that importer with jalapeño and serrano peppers grew the outbreak strain.

**Conclusions/Significance:**

Jalapeño peppers, contaminated before arrival at the restaurants and served in uncooked fresh salsas, were the source of these infections. Our investigations, critical in understanding the broader multistate outbreak, exemplify an effective approach to investigating large foodborne outbreaks. Additional measures are needed to reduce produce contamination.

## Introduction

Long-distance transportation keeps stores and restaurants stocked with a wide assortment of fresh fruits and vegetables year round. However, with this abundance has come increased risk of large produce-associated foodborne outbreaks [1]–[3]. Among produce-associated outbreaks with known etiologic agents, the most common pathogen is non-typhoidal *Salmonella*, a diverse group of bacteria that live in intestinal tracts of many animals and survive in environments from the farm to the table [3], [4]. The overall incidence of *Salmonella* infection from all sources in 2009 was more than double the national target for 2010; little progress towards this goal has been observed since 1996 [5].

In May 2008, a multistate outbreak of *Salmonella enterica* serotype Saintpaul infections with indistinguishable pulsed-field gel electrophoresis (PFGE) patterns was detected which ultimately became the largest known foodborne outbreak in the United States in over 10 years. Initial investigations identified an association between illness and the consumption of raw tomatoes [6]. On June 7, the US Food and Drug Administration (FDA) issued a national alert to avoid “raw red plum, red Roma, and red round tomatoes” [7].

As cases continued to occur, a specific variety of tomato could not be epidemiologically implicated and the sources of suspected tomatoes were not converging to any specific growing region or to a common distributor. Furthermore, a possible association between infections and eating Mexican-style foods began to emerge. Because tomatoes are a staple ingredient in many cuisines, not just Mexican, this finding suggested there might be something else, besides tomatoes, more unique to Mexican food involved. Collectively, these observations raised concern that an unidentified vehicle of infection might exist.

In mid-June, a cluster of cases was identified among persons who ate at Restaurant A, a Mexican-style restaurant in Wichita Falls, Texas, providing an opportunity for evaluation of the discrete list of foods served. A second cluster of cases was subsequently identified among persons who ate at two locations of Mexican-style Restaurant Chain B, in northern Texas. Here we describe the investigation of cases associated with Restaurant A and Restaurant Chain B conducted to further characterize the source of infections in the broader multistate outbreak.

## Methods

### Ethics statement

The National Center for Emerging and Zoonotic Infectious Diseases within the Centers for Disease Control and Prevention determined that these investigations did not meet the definition of research as provided by 45 CFR4 6.102(d) and therefore IRB review was not required. The basis for this determination was that the primary purpose of this activity was to identify, characterize, and control disease in response to an immediate public health threat. All participants were explained the purpose of the investigation and participation was voluntary.

### Case definitions

We defined confirmed cases as culture-confirmed *Salmonella* infections reported to the Texas Department of State Health Services (TXDSHS) or Wichita Falls-Wichita County Public Health District (WFWCPHD) during June 2008 in persons who ate food from Restaurant A or from either of two Restaurant Chain B establishments during the seven days before diarrhea began. We defined probable cases as ≥3 loose stools in a 24-hour period starting within 7 days after eating at Restaurant A or at either Restaurant Chain B location.

### Case finding

We obtained lists of culture-confirmed *Salmonella* infections in each of the three counties where the three restaurants were located. Outbreak strain infections, defined as *Salmonella enterica* serotype Saintpaul with *Xba*I PFGE pattern JN6X01.0048, were detected through routine laboratory-based surveillance, whereby clinical laboratories forward *Salmonella* isolates to public health laboratories for serotyping, PFGE analysis, and reporting [6]. Additional culture-confirmed *Salmonella* infections for which subtyping analyses were not performed were detected through mandated *Salmonella* case-based reporting by physicians.

We then determined which infections occurred in persons who had consumed food from Restaurant A or from either Restaurant Chain B establishment during the 7 days before diarrhea began through telephone interviews. These confirmed case-patients were asked to provide names and telephone numbers of their meal companions. Probable case-patients were identified from this list of meal companions. Several probable case-patients with resolved gastroenteritis submitted rectal swabs for culture at the TXDSHS laboratory during our investigation to more accurately classify case-patients.

### Case-control studies

Beginning June 21, we conducted sequential, independent, matched case-control studies of Restaurant A and Restaurant Chain B patrons. Well meal companions, defined as persons who ate meals at either Restaurant A or B with confirmed case-patients but who did not develop diarrhea, abdominal cramping, fever, or vomiting during the 7 days following the meal, were enrolled as controls. Trained interviewers administered standardized questionnaires developed to measure exposure to all menu items. Because patrons could modify menu items by asking that standard ingredients or condiments be excluded or by adding non-standard ingredients or condiments, respondents were systematically questioned about any modifications made. In addition to assessing exposures to menu items, we obtained recipes for each item, to assess exposures to specific ingredients.

### Statistical analysis

Data from each case-control study were separately analyzed using SAS 9.2 (SAS Institute, Cary, NC). We used exact conditional logistic regression to obtain matched odds ratios and 95% confidence intervals for the association between illness and each menu item and ingredient. Because both gender and age are often associated with diet preferences, we also calculated sex- and age group- (<10 and ≥10 years) adjusted estimates. We used a p-value threshold of ≤0.05 for significance and did not adjust for multiple comparisons. Not all case-patients had consumed their meals with controls. Therefore, to include as many case-patients as possible in the analyses, we matched case-patients to controls by meal date. We allowed a variable ratio of controls to cases in each matched set. Sex- and age group-adjusted multivariate models were constructed when two or more menu items or ingredients were associated with illness in univariate analysis. To maximize our sensitivity to detect an association between raw tomato consumption and illness, all menu items that regularly contained raw tomatoes were treated as containing them even if the restaurant reportedly stopped using raw tomatoes in response to initial outbreak alerts.

### Restaurant environmental investigations

Environmental investigations of the restaurants included review of food storage and preparation, inquiries into worker absenteeism and illness, and collection of environmental and food samples for testing using the FDA's Bacteriological Analytical Manual (BAM) *Salmonella* culture method at TXDSHS and WFWCPHD laboratories [8].

### Traceback investigation

The FDA and Centers for Disease Control and Prevention (CDC) formed a joint team in July 2008 to trace the path of implicated product from both Restaurant A and Restaurant Chain B back to the farm. We used dates of patient exposures to the restaurants as the starting point to identify suspect shipments. Distribution records, including invoices and bills of lading, were used to determine shipment and receipt dates at each point in the supply chain. FDA investigators collected product and environmental samples for testing using the BAM method at FDA laboratories [8].

## Results

### Restaurant A epidemiological investigation

#### Case finding

Thirty-two culture-confirmed outbreak strain or non-subtyped *Salmonella* infections were identified during June 2008 in Wichita County. Of 30 patients who could be contacted, 29 (97%), met the confirmed case definition and consumed food from Restaurant A. In addition, 25 probable case-patients were identified; three submitted rectal swabs and were reclassified as confirmed case-patients following isolation of the outbreak strain. All patients dined at Restaurant A from May 30 through June 2.

#### Case-control study

We enrolled 47 case-patients (25 confirmed, 22 probable). Of the 25 confirmed case-patients, 19 had outbreak strain infections; *Salmonella* isolates from the other six were not sent to the TXDSHS laboratory for serotyping or subtyping. We enrolled 40 meal date-matched controls.

**Figure 1:**
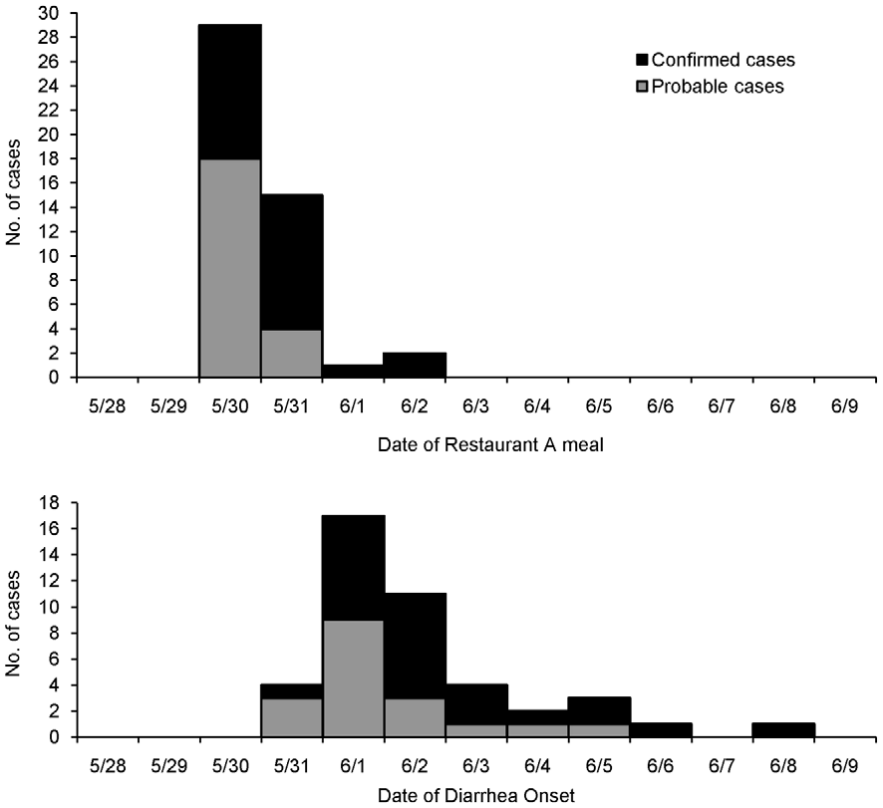
Restaurant A-associated cases by date of meal (top) and diarrhea onset (bottom). Black bars represent 25 confirmed cases. Grey bars represent 22 probable cases. The exact dates of diarrhea onset are not available for 4 probable cases.

Among enrolled case-patients, dates of dining at Restaurant A and dates of diarrhea onset were similar for culture-confirmed and probable cases (Figure 1). The median incubation period was 2 days for both culture-confirmed (range, <1–7 days) and probable (range, 1–5 days) cases. Case-patients ranged in age from 2 to 63 years; 51 percent were female, four (9%) were hospitalized, and none died.

Of 244 menu items individually evaluated, only red salsa and cheese sauce were statistically associated with illness. Red salsa was consumed by 45 (96%) case-patients and 11 (28%) controls (matched odds ratio [mOR], 47; 95% confidence interval [CI], 9.9–456). Cheese sauce was consumed by 43 (91%) case-patients and 28 (70%) controls (mOR, 4.4; 95% CI, 1.2–21) (Table 1). In a multivariate model containing red salsa and cheese sauce as independent exposure variables, only red salsa was independently associated with illness (adjusted-mOR, 37; 95% CI, 7.2–386) (Table 2). Of ingredients individually evaluated, consumption of raw jalapeño peppers and raw tomatoes as ingredients in any dish were statistically associated with illness. Forty-five (96%) case-patients and 16 (40%) controls ate raw jalapeño peppers (mOR, 28; 95% CI, 6.1–267). Forty-six (98%) case-patients and 29 (73%) controls ate raw tomatoes (mOR, 18; 95% CI, 2.3–833) (Table 1). In a multivariate model containing raw tomatoes and raw jalapeño peppers as independent exposure variables, only raw jalapeño peppers were independently associated with illness (adjusted-mOR, 25; 95% CI, 3.4–>1000) (Table 2). Adjustment for age and sex did not alter findings of the Restaurant A case-control study (Table 1).

**Table 1:** Consumption of selected food items by case-patients and controls by restaurant cluster. ^a^

	Patients		Controls		Matched Odds Ratio		Adjusted Matched Odds Ratio^b^	
**Restaurant A**	**(N = 47)**		**(N = 40)**		**(95% CI)**		**(95% CI)**	
*Menu item consumed*		*no (%)*						
Red salsa	45	(96)	11	(28)	47	(9.9–456)^c^	42	(8.7–413)^c^
Cheese sauce	43	(91)	28	(70)	4.4	(1.2–21)^c^	4.7	(1.2–23)^c^
Tortilla chips	41	(87)	33	(83)	1.4	(0.4–5.4)	1.4	(0.4–5.5)
Guacamole	30	(64)	21	(53)	1.7	(0.6–4.6)	1.6	(0.6–4.5)
Fajitas	20	(43)	23	(58)	0.4	(0.1–1.4)	0.3	(0.1–1.4)
*Ingredient consumed*		*no (%)*						
Raw tomatoes	46	(98)	29	(73)	18	(2.3–833)^c^	15	(1.9–714)^c^
Raw jalapeño peppers	45	(96)	16	(40)	28	(6.1–267)^c^	24	(5.2–226)^c^
Avocados	30	(64)	21	(53)	1.7	(0.6–4.6)	1.6	(0.6–4.5)
**Restaurant Chain B**	**(N = 21)**		**(N = 31)**					
*Menu item consumed*		*no (%)*						
Red salsa	21	(100)	24	(77)	8.1^d^	(1.0–∞)	13^d^	(1.3–∞)^c^
*Ingredient consumed*		*no (%)*						
Raw jalapeño peppers	21	(100)	25	(81)	7.7^d^	(0.9–∞)	13^d^	(1.3–∞)^c^
Raw tomatoes	15	(71)	16	(52)	1.7	(0.4–7.8)	1.7	(0.4–8.7)

a Exposures reported by at least 40 percent of case-patients are shown.

b Adjusted for sex and age group (<10 versus ≥10 years old).

c p-value <0.05.

d Median unbiased estimate.

**Table 2:** Multivariate assessment of associations of menu items and ingredients with illness among Restaurant A patrons.

	Patients		Controls		Adjusted Matched Odds Ratio^a^	
Restaurant A	(N = 47)		(N = 40)		(95% CI)	
*Menu Item consumed* b		*no (%)*				
Red salsa	45	(96)	11	(28)	37	(7.2–386)^c^
Cheese sauce	43	(91)	28	(70)	0.9	(0.1–8.5)
*Ingredient consumed* d		*no (%)*				
Raw tomatoes	46	(98)	29	(73)	1.0	(0.01–81)
Raw jalapeño peppers	45	(96)	16	(40)	25	(3.4–>1000)^c^

a Adjusted for sex and age group (<10 versus ≥10 years old).

b Red salsa and cheese sauce are the two exposure variables in the menu item model.

c p-value <0.05.

d Raw tomatoes and raw jalapeño peppers are the two exposure variables in the ingredient model.

### Restaurant chain B epidemiological investigation

#### Case finding

Thirty-three culture-confirmed outbreak strain or non-subtyped *Salmonella* infections were identified through routine surveillance during June 2008 in the two counties in which the Restaurant Chain B establishments were located. Of 32 patients who could be contacted, 23 (72%) met the confirmed case definition and consumed food from either of the two Restaurant Chain B establishments. In addition, nine probable case-patients were identified. All patients dined at Restaurant Chain B from May 23 through June 12, with only two exposures before June 2.

#### Case-control study

**Figure 2:**
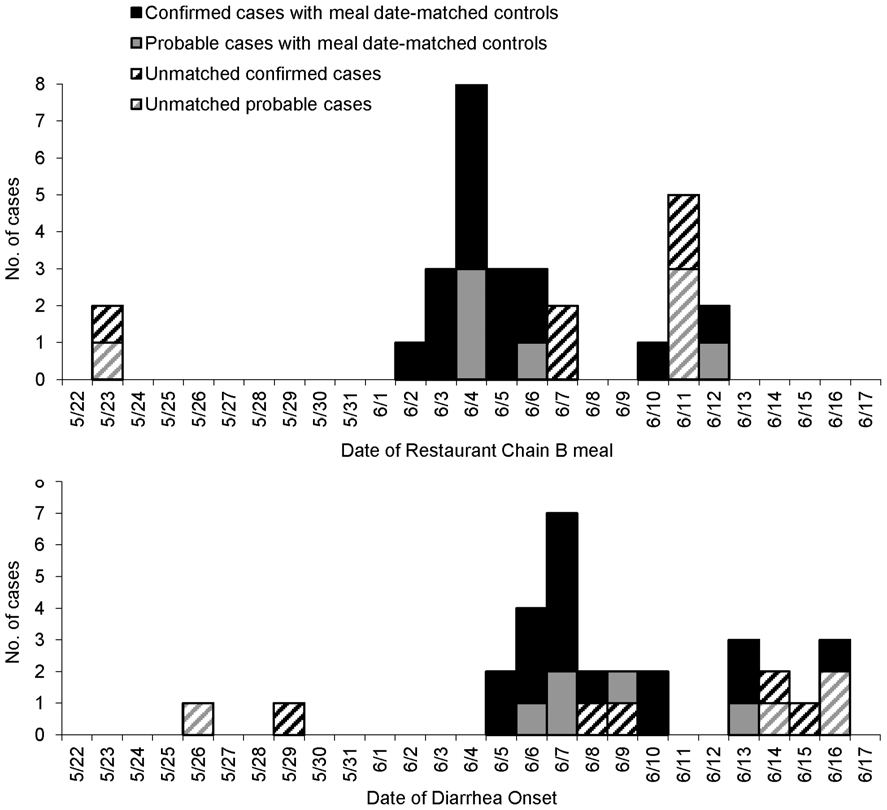
Restaurant B-associated cases by date of meal (top) and diarrhea onset (bottom). Solid black bars represent 16 confirmed cases with meal date-matched controls. Solid grey bars represent 5 probable cases with meal date-matched controls. The dashed black and grey bars represent 5 confirmed and 4 probable cases, respectively, that were not included in the matched analyses because they had no meal date-matched controls.

We enrolled 30 case-patients (21 confirmed, 9 probable). Of the 21 confirmed case-patients, 13 had outbreak strain infections; *Salmonella* isolates from the other eight were not sent to the TXDSHS laboratory for serotyping or subtyping. We enrolled 30 meal date-matched controls. Matched controls could not be found for nine (5 confirmed, 4 probable) case-patients, leaving only 21 case-patients included in the matched analyses (Figure 2).

Among enrolled case-patients, dates of dining at Restaurant Chain B and dates of diarrhea onset were similar for culture-confirmed and probable cases (Figure 2). The median incubation period was 3 days for both culture-confirmed (range, 1–7 days) and probable (range, 1–5 days) cases. Case-patients ranged in age from 5 to 76 years; 47 percent were female, nine (30%) were hospitalized, and none died.

Of 101 menu items individually evaluated, no items were significantly associated with illness in the absence of adjustment for possible confounders. With adjustment for age and sex, only red salsa was statistically associated with illness, consumed by all 21 case-patients and 24 (77%) controls (adjusted-mOR, 13; 95% CI, 1.3–infinity) (Table 1). No ingredients were significantly associated with illness in the absence of adjustment for possible confounders. With adjustment for age and sex, the only ingredient in Restaurant Chain B dishes associated with illness was raw jalapeño peppers, consumed by all 21 case-patients and 25 (81%) controls (adjusted-mOR, 13; 95% CI: 1.3–infinity) (Table 1). All nine case-patients excluded for lack of meal date-matched controls consumed red salsa and raw jalapeño peppers at the restaurants.

### Environmental findings

The Restaurant A salsa contained raw Roma or red round tomatoes, raw jalapeño peppers, salt, granulated garlic, and red pepper flakes. During the 4 day period in which case-patients ate at Restaurant A, approximately 25 25-pound (11.3 kg) boxes of tomatoes of various brands were used by the restaurant, whereas only one box of jalapeño peppers was likely used.

The recipe for Restaurant Chain B salsa was similar to Restaurant A's, except that it always used commercially canned tomatoes, not raw tomatoes. Restaurant A and Restaurant Chain B used different brands of salt, granulated garlic, and red pepper flakes. Therefore, the only ingredient that might have been in common between the two salsas was raw jalapeño peppers. The dry spices were purchased in bulk, lasting longer than the observed exposure periods.

The implicated salsas were made in large batches. Individual servings were placed on multiple trays stacked above a single layer of ice in the service lines. The restaurants did not routinely measure the temperature of the salsa on these trays.

The restaurant managers denied any food worker illness or absenteeism during the case-patients' exposure periods. Cultures of food and environmental samples, including tomatoes, jalapeño peppers, salsa, spices, and swabs of food processing and storage surfaces collected from Restaurant A and one Restaurant Chain B location did not yield serotype Saintpaul. However, no foods served during the dates of exposure were available for testing.

### Traceback investigation

Jalapeño peppers used in Restaurant A during the period of case-patient exposure were traced to Importer A in southern Texas. Jalapeño peppers used in the Restaurant Chain B restaurants during the period of case-patient exposure were traced to Importer B, located near Importer A. This area of Texas is home to many produce import firms. During our record review we directly observed that these firms commonly trade goods amongst themselves to fill orders.

**Figure 3:**
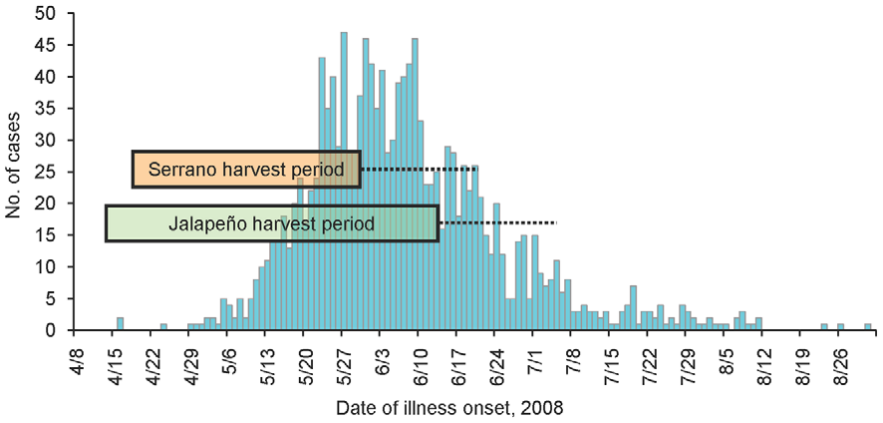
Cases by date of onset in multistate outbreak and dates of pepper harvest. The blue bars represent 1500 cases in the multistate outbreak of *Salmonella* Saintpaul infections reported from 43 states and the District of Columbia, and Canada; these data are modified from reference 6. The orange bar represents the period of serrano pepper harvest (April 18 through May 31) on the farm from which the outbreak strain was isolated. The green bar represents the period of jalapeño pepper harvest (April 14 through June 14) on the same farm. Jalapeño and serrano peppers might begin to wrinkle and lose quality as early as three weeks after harvest (indicated by dashed lines to the right of harvest periods), but refrigeration might extend their shelf life well beyond three weeks (FDA, personal communication).

Culture of a jalapeño pepper sample collected from Importer B on July 11 yielded the outbreak strain. These peppers were traced to a packing facility in Nuevo Leon, Mexico. The traceback from the packing facility was complex with commingling of product and a network of interrelated distribution points. FDA investigations continued on two Mexican farms (Farm A and Farm B) that were major suppliers of peppers to the packing facility, though records indicate that other farms also supplied the packing facility during this time period. Farm A grew Roma tomatoes in addition to jalapeño and serrano peppers. Environmental sampling at Farm A identified *Salmonella,* but none that were serotype Saintpaul. Farm B, located approximately 100 miles from Farm A, was the packing facility's main pepper supplier. It grew jalapeño peppers and serrano peppers, but not tomatoes, and harvested produce from mid-April to mid-June (Figure 3). The outbreak strain was isolated from two samples taken from Farm B—agricultural water and serrano peppers found in the field.

## Discussion

In the midst of a massive multistate outbreak of *Salmonella* serotype Saintpaul infections, investigation of two clusters of cases among patrons of restaurants in Texas provided evidence implicating jalapeño peppers. First, the only ingredient in the restaurants independently associated with illness was raw jalapeño peppers. Second, the only ingredient in common between the two restaurant salsa recipes was raw jalapeño peppers. Third, the outbreak strain was isolated from serrano peppers and irrigation water on a farm that grew epidemiologically implicated jalapeño peppers. Harvesting of peppers from the farm began shortly before the first cases of the multistate outbreak and continued for a duration that could account for the entire outbreak. Although, to our knowledge, no reports of *Salmonella* infections acquired from consumption of jalapeño peppers existed before our investigations, it was known that *Salmonella* grows well in extracts of homogenized jalapeño peppers [9].

This outbreak is one in a series of increasingly recognized, large, and widely-dispersed outbreaks caused by contaminated produce [10]–[17]. The proportion of all reported foodborne outbreaks in the United States associated with contaminated produce increased from 0.7% in the 1970s to 6% in the 1990s; this trend appears to be ongoing [2], [3]. Related forces likely driving this shift include increasing consumption of fresh produce, increasing centralization of the produce industry to maintain year-round availability, expansion of growing fields to areas adjacent to animal production areas, and improved detection and investigation of widely dispersed outbreaks [1], [2], [18]. Centralization might foster outbreaks by increasing the number of points at which contamination may occur and the amount of product that could be contaminated in a given event. Centralization could also lead to more widely dispersed outbreaks because contaminated produce may be widely distributed [19].

Routine laboratory-based surveillance, especially molecular subtyping by PulseNet USA, has greatly improved our ability to detect widely dispersed outbreaks [20]. However, investigation of these outbreaks can be challenging when there is little apparent epidemiologic clustering by person, place, or time, and because of time delays inherent with laboratory-based surveillance. When generating hypotheses, investigators must consider an enormous list of possible food, water, animal, and environmental exposures [2]. Furthermore, patients are often interviewed several weeks after becoming ill, when recall of basic foods consumed is limited, and even more diminished for specific details, such as tomato type or ingredients within prepared dishes.

Our investigations highlight how these challenges can be reduced by searching for and investigating clusters of cases associated with specific food establishments or events within a larger outbreak. Investigations of localized clusters simplify hypothesis generation because the suspected sources of infection are usually limited to a finite list of menu items [2]. Likewise, patient recall is enhanced by focusing on a specific, often memorable, meal. Furthermore, investigators can obtain recipes to assess associations between illness and specific ingredients; this is especially helpful in identifying stealthy vehicles, such as jalapeño peppers, that some people might have been unaware they consumed. Additionally, turnover rate of ingredients within restaurants can be evaluated to find those ingredients that best fit the exposure period of patients in the outbreak. For example, at Restaurant A we noted that approximately 25 boxes of tomatoes would have been used during the 4-day period when patients ate there compared with one box of jalapeno peppers; thus contamination present in just one box of peppers could account for the Restaurant A outbreak duration. Finally, food delivery invoices kept by restaurants and caterers contain specific information that can improve the accuracy of traceback investigations; this is particularly important in fresh produce-associated outbreaks because fruits and vegetables sold in stores typically come in many varieties and may have minimal labeling to identify their source. Collectively, these attributes of localized cluster investigations serve to generate more specific exposure information [21].

Detailed exposure information allowed us to document the stealthy nature of jalapeño peppers in this outbreak. Three case-patients in our study of Restaurant Chain B also participated in a multistate case-control study [6]. Our study, aided by restaurant recipes, documented that all three consumed foods containing raw jalapeño peppers at the restaurant, as all reported having consumed salsa; one patient also reported adding raw jalapeño peppers to an entrée. In the multistate study, which asked patients to recall consumption of specific ingredients in restaurants, the two patients, whose only exposure to raw jalapeño peppers at Restaurant Chain B was from salsa, denied exposure to this ingredient, presumably because they were unaware that it was present in the salsa.

Some restaurant-prepared fresh salsas might be prone to amplifying and spreading small amounts of bacterial contamination present on a few individual produce items to a large number of servings because they often are made in large batches containing pooled and diced raw produce ingredients [21]. *Salmonella* grows better on diced, as compared with intact, tomatoes and jalapeño peppers [22]. Diced tomatoes, and any foods containing them, unless acidified to a pH of <4.2, are included in the 2009 FDA Food Code as a potentially hazardous food, and thus require storage at or below 41°F (5°C) [23]. High *Salmonella* growth rates have been observed in salsas prepared using the Restaurant A and Restaurant Chain B recipes at temperatures at or above 54°F (12°C) [22]. Growth might be reduced by replacing granulated garlic with fresh garlic and adding lime juice to the recipes [22].

Because so much produce is consumed raw and disinfection methods are not highly effective on fresh produce, the keys to preventing produce-associated outbreaks are preventing the initial contamination and minimizing handling practices that lead to amplification [2]. Water used for irrigation and pesticide application is one possible source of contamination in this outbreak. Keeping water used for these purposes protected from animals and waste run-off is important. Modifying salsa recipes to include growth-inhibitory ingredients might limit amplification of *Salmonella* [22]; all fresh salsas with a pH ≥4.2 should be stored in adherence with established time and temperature recommendations to minimize growth [24]. Additionally, because *Salmonella* grows rapidly in diced jalapeño peppers, regulatory consideration is warranted to define foods containing them as potentially hazardous.

During the summer of 2008, 1,500 *Salmonella* serotype Saintpaul infections with the outbreak PFGE pattern were reported from 43 states and the District of Columbia and Canada. Shortly after jalapeño peppers were identified as the probable source of infections in the two Texas clusters described in this report, an independent investigation of restaurant-acquired serotype Saintpaul infections in Minnesota implicated raw jalapeño peppers [6]. During the outbreak, 33 restaurant clusters were reported nationally; 31 served foods that contained jalapeño or serrano peppers [6].

The Farm B chili pepper harvest period (April 14–June 14) closely mirrors the range of illness onset dates in the multistate outbreak (April 16–August 26) (CDC and FDA, unpublished data). Chili peppers might begin to wrinkle and lose quality 3 weeks after harvest, but refrigeration may extend their shelf life well beyond this period (FDA, personal communication). Therefore, Farm B peppers were harvested shortly before and available during this multistate outbreak (Figure 3). On July 30, national alerts advised persons to avoid raw jalapeño and serrano peppers grown or packed in Mexico. The two restaurant cluster investigations in Texas reported here were critical in solving this complex multistate outbreak.
